# Congenital Cataract Causing Mutants of αA-Crystallin/sHSP Form Aggregates and Aggresomes Degraded through Ubiquitin-Proteasome Pathway

**DOI:** 10.1371/journal.pone.0028085

**Published:** 2011-11-29

**Authors:** Ilangovan Raju, Edathara C. Abraham

**Affiliations:** Department of Biochemistry and Molecular Biology, University of Arkansas for Medical Sciences, Little Rock, Arkansas, United States of America; National Institutes of Health, United States of America

## Abstract

**Background:**

Mutations of human αA-crystallin cause congenital cataract by protein aggregation. How mutations of αA-crystallin cause disease pathogenesis through protein aggregation is not well understood. To better understand the cellular events leading to protein aggregation, we transfected cataract causing mutants, R12C, R21L, R21W, R49C, R54C, R116C and R116H, of human αA-crystallin in HeLa cells and examined the formation of intracellular protein aggregates and aggresomes by confocal microscopy.

**Methodology/Principal Findings:**

YFP-tagged human αA-wild-type (αA-wt) was sub-cloned and the mutants were generated by site-directed mutagenesis. The αA-wt and the mutants were individually transfected or co-transfected with CFP-tagged αA-wt or αB-wild-type (αB-wt) in HeLa cells. Overexpression of these mutants forms multiple small dispersed cytoplasmic aggregates as well as aggresomes. Co-expression of αB-wt with these mutants significantly inhibited protein aggregates where as co-expression with αA-wt enhanced protein aggregates which seems to be due to co-aggregation of the mutants with αA-wt. Aggresomes were validated by double immunofluorescence by co-localization of γ-tubulin, a centrosome marker protein with αA-crystallin. Furthermore, increased ubiquitination was detected in R21W, R116C and R116H as assessed by western blot analyses. Immunostaining with an ubiquitin antibody revealed that ubiquitin inclusions in the perinuclear regions were evident only in R116C transfected cells. Pulse chase assay, after cycloheximide treatment, suggested that R116C degraded faster than the wild-type control.

**Conclusions/Significance:**

Mutants of αA-crystallin form aggregates and aggresomes. Co-expression of αA-wt with the mutants increased aggregates and co-expression of αB-wt with the mutants significantly decreased the aggregates. The mutant, R116C protein degraded faster than wild-type control and increased ubiquitination was evident in R116C expressing cells.

## Introduction

Cataract of the eye lens is the leading cause of blindness worldwide [1]. Pediatric cataract of the congenital type is the most common form of childhood blindness and it is clinically and genetically heterogeneous. About 30-50% of all bilateral pediatric cataracts have a genetic basis [2]. All three forms of Mendelian inheritance have been observed, the most frequently observed type seen in non-consanguineous population being the autosomal dominant transmission. At least 34 loci in the human genome have been reported to be associated with various forms of pediatric cataracts. Autosomal dominant and recessive forms of cataracts have been caused by mutations in 22 different genes [2]. More than half of the mutations occur in crystallins (α-, β-, and γ-crystallins) and the remaining in connexins, intrinsic membrane proteins and intermediate filament proteins. Most interestingly, a total of 12 mutants belong to α-crystallin, 8 for αA-crystallin and 4 for αB-crystallin., it points to a major role α-crystallin mutants play in the development of congenital cataracts.

The α-crystallin gene family consists of two similar genes coding for αA-crystallin, CRYAA located on chromosome 21q22.3, and for αB-crystallin, CRYAB located on chromosome 11q22.1 [3]. The first exon of each gene encodes 60 amino acids consisting of a repeat of 30 amino acid motif and the second and the third exons code for regions homologous for the sHsps [4]. Three αA-crystallin missense mutations have been reported recently which are: base 104 C>T (R12C), base 130 C>T (R21W) and base 230 C>T (R54C) [5]. The affected members of the three families had autosomal dominant bilateral congenital nuclear cataract in association with microcornea, all detected at the time of birth. Affected members of one of this family (R21W) were also diagnosed with microphthalmia. R12C and R21W cases showed zonular opacification with varying involvement of the anterior and posterior pole. It is noteworthy that these mutations occurred outside the α-crystallin/sHsp core domain. Moreover, the arginines at positions 12, 21, and 54 are highly conserved in αA-crystallin. The other αA mutants reported earlier with autosomal dominant congenital cataracts are: R21L [6], R49C [7], G98R [8], R116C [9] and R116H [10].

A recent report [11] on the biophysical as well as the hydrodynamic properties of the mutants of αA-crystallin have prompted us to further investigate the actual mechanism by which these mutations can lead to early onset of cataract. In all the seven mutants, arginine residues were mutated to mostly cysteine, leucine, tryptophan or histidine. In this study [11], the quaternary structural parameters (hydrodynamic properties) were determined by dynamic light scattering measurements. As compared to αA-wt, average molar mass, polydispersity, and hydrodynamic radius increased several fold in R116C and R116H, moderately increased in R12C, R21W, and R54C, and not increased in R21L and R49C. With regard to secondary and tertiary structural changes, all the mutants showed varying degree of secondary and tertiary structural changes, R21W, R116C, and R116H consistently showing the largest changes. Such changes can lead to protein unfolding/misfolding and subsequently forming protein aggregates.

Since mutants of αA-crystallin contribute to the development of congenital cataract through the formation of aggregated proteins precipitated in the cells of eye lens, we evaluated the expression of mutants of αA-crystallin in mammalian cells (HeLa cells) in terms of identifying the cells having aggregates and aggresomes as the general cellular response to having over expressed mutant proteins [12]. Aggresomes are thought to immobilize protein aggregates and render them susceptible to proteolysis by a component known as proteasomes and/or autophagy [13]]–[[15]. In view of the propensity of αA-crystallin mutants to aggregate in cells, we also explored whether there is any involvement of ubiquitin-proteasomal pathway (UPP) contributing to the degradation of unfolded proteins of mutants of αA-crystallin. The ubiquitin-proteasome system (UPS) plays an essential role in degrading damaged or unfolded proteins [16]. Unfolded proteins and protein fragments generated by proteolysis are polyubiquitinated by ubiquitin ligases, a process that targets the substrate proteins to the proteasome for degradation [17]. The 26S proteasome which consists of a catalytic 20S core particle and a 19S regulatory particle selectively degrades ubiquitinated proteins [18]; our results suggest that the mutants, R21W, R116C and R116H have elevated polyubiquitinated species.

## Materials and Methods

### Site directed mutagenesis

To generate mutants, QuickChange site directed mutagenesis kit (Agilent Technologies Inc, CA) was used. Appropriate mutagenic primers of human αA-crystallin for the mutants, R12C, R21L, R21W, R49C, R54C, R116C and R116H were designed and used for PCR. The PCR products were amplified by using YFP-tagged αA-wt as a template DNA with the following PCR conditions, the mix was initially denatured at 95°C for 1 min followed by 95°C for 50 sec, 60°C for 50 sec and 68°C for 5 minutes for 16 cycles and followed by overall extension at 68°C for 7 minutes. The PCR product was digested with *Dpn* I for 1 hour at 37°C and 1 µl of PCR product was transformed with XL-10 Gold competent cells. The transformants were selected on LB agar medium plates containing 50 µg/ml Kanamycin. The mutant constructs were sequenced and confirmed by DNA sequence analysis. Untagged pCDNA3.1-αA-wt and the mutant R116C were PCR amplified from appropriate cDNA templates and sub-cloned into *Xho* I and *Eco* RI sites of pCDNA3.1 (-) vector (Invitrogen, CA). The constructs were validated by restriction digestion and DNA sequence analyses.

### Cell culture and transfection

HeLa cells purchased from ATCC, Manassas, VA were grown in 35 mm dishes and 80–90% confluent cells were transfected with Lipofectamine 2000 (Invitrogen, CA) and a total of 2 µg of plasmid DNA encoded for αA-wt and αB-wt and or mutated constructs fused with either CFP or YFP were used. In one set of experiments, individual constructs for αA-wt and the mutants and for co-expression studies, equal amount of both αA-wt and αB-wt with mutated αA constructs were transfected. Transfected cells showing aggregates were typically counted at x40 magnification. Fields were randomly chosen and about 300 cells were counted per experiment and repeated at least three times and counts were blindly performed.

### Laser scanning confocal microscopic studies

An LSM 510 Laser Scanning Microscope (Carl Zeiss Inc., Thornwood, NY) with 63x oil-immersion objective (plan Apochromat, NA 1.4) (University of Arkansas for Medical Sciences core facility) was utilized. To visualize CFP and YFP fluorescence, cells expressing fluorescent proteins were excited at appropriate laser beam and filtered with both dichromatic band pass filters, captured at 12 bit 512 x 512, multitrack channel images with CCD cameras with the following configurations: for CFP channel, the cells were excited with 458 nm filter by argon-ion laser and the emission intensity was collected using band pass (BP) 475-525 nm filters and for YFP channel, the cells were excited with 514 nm filter by argon-ion laser and the emission intensity was collected using BP 530–600 nm filters. Both the CFP and YFP was excited using argon-ion laser at 25 mW, 2.0 and 0.5% exposure respectively.

### Cycloheximide chase assay

Degradation of αA wild-type and the mutant αA-R116C proteins was assessed using cycloheximide-chase assay. HeLa cells were grown in 35 mm dishes and transfected with untagged αA-wt and the mutant R116C. After 24 h transfection, cells were treated with 20 µg/mL of cycloheximide (Sigma) for the indicated time period and lysed with lysis buffer containing 50 mM Tris-HCl (pH 7.4), 150 mM NaCl, 0.02% sodium azide, 0.1% SDS, 1% NP-40, 0.5% sodium deoxycholate and 0.1 mM EDTA supplemented with cock-tail protease inhibitors (Roche Diagnostics) and 3M Urea. For immunoblot analysis, 5 µg of total protein was loaded into 12% SDS-PAGE and the western blot was probed with a rabbit polyclonal anti-αA-crystallin antibody (Enzo Life Sciences Inc, SPA-221) at a dilution of 1 in 6000.

### SDS-PAGE and Western blot analysis

After 48 hours transfection, cells were lysed with lysis buffer containing 50 mM Tris-HCl (pH 7.4), 150 mM NaCl, 0.02% sodium azide, 0.1% SDS, 1% NP-40, 0.5% sodium deoxycholate and 0.1 mM EDTA supplemented with cock-tail protease inhibitors and 3M Urea. Further, the cell lysate was sonicated and the protein concentration was measured by BCA assay method. For each sample, 5 µg of total protein was loaded into 12% SDS-PAGE and electroblotted to PVDF membrane. The blots were blocked with 5% non-fat dry milk prepared in TBST (Tris-buffered saline supplemented with 0.1% Tween 20) and subsequently incubated with primary antibody for αA-crystallin (monoclonal, Abcam, ab78439, 1∶2000), αB-crystallin (rabbit polyclonal, Abcam, ab13497, 1∶2000) for one hour at room temperature. Blots were washed with TBST for three times and incubated with appropriate HRP-conjugated secondary antibodies (1 in 10000, Santa Cruz Biotechnology Inc, CA) for one hour at room temperature. Enhanced Chemiluminescence substrate was used and the signal was detected by exposing the blots on films. For loading control, blots were stripped with Restore Western Blot stripping buffer (Thermo Scientific Inc, IL) and re-probed with a rabbit polyclonal antibody against β-actin (Abcam, ab8227, 1∶10000) for 1 hour at room temperature.

### Immunofluorescence microscopy for aggresome detection

Cells were grown on 35-mm cover glass bottom dishes. After 48 hours transfection, cells were washed with PBS, fixed with 4% paraformaldehyde for 20 minutes at room temperature (RT) and permeabilized with 0.5% Triton X-100 for 10 minutes at RT. Cells were blocked with 5% normal goat serum (NGS) for one hour at RT. The cells were simultaneously incubated with αA-crystallin mouse monoclonal antibody (1 in 200 in 5% NGS, Abcam, ab78439) and a rabbit polyclonal antibody for γ-tubulin (Abcam, ab16504; 1 in 200 dilution in 5% NGS) for overnight at 4°C. The cells were stained with Alexa Flour 594 (mouse) and Alexa Fluor 488 (rabbit) (Molecular Probes) for one hour at room temperature. Nuclei were counter stained with Hoechst 33342. The images were acquired with an LSM 510 Meta Carl Zeiss Confocal microscope at x63 objective and analyzed using AIM Imaging Software.

### Data Analysis and Statistics

In all the experiments, values were expressed as mean ± SD. Two-tailed Student's t-test was used for statistical analysis. The p value < 0.05 was considered as significant.

## Results

### Individual expression of YFP-tagged αA-crystallin wild-type and mutants in HeLa cells

**Figure 1:**
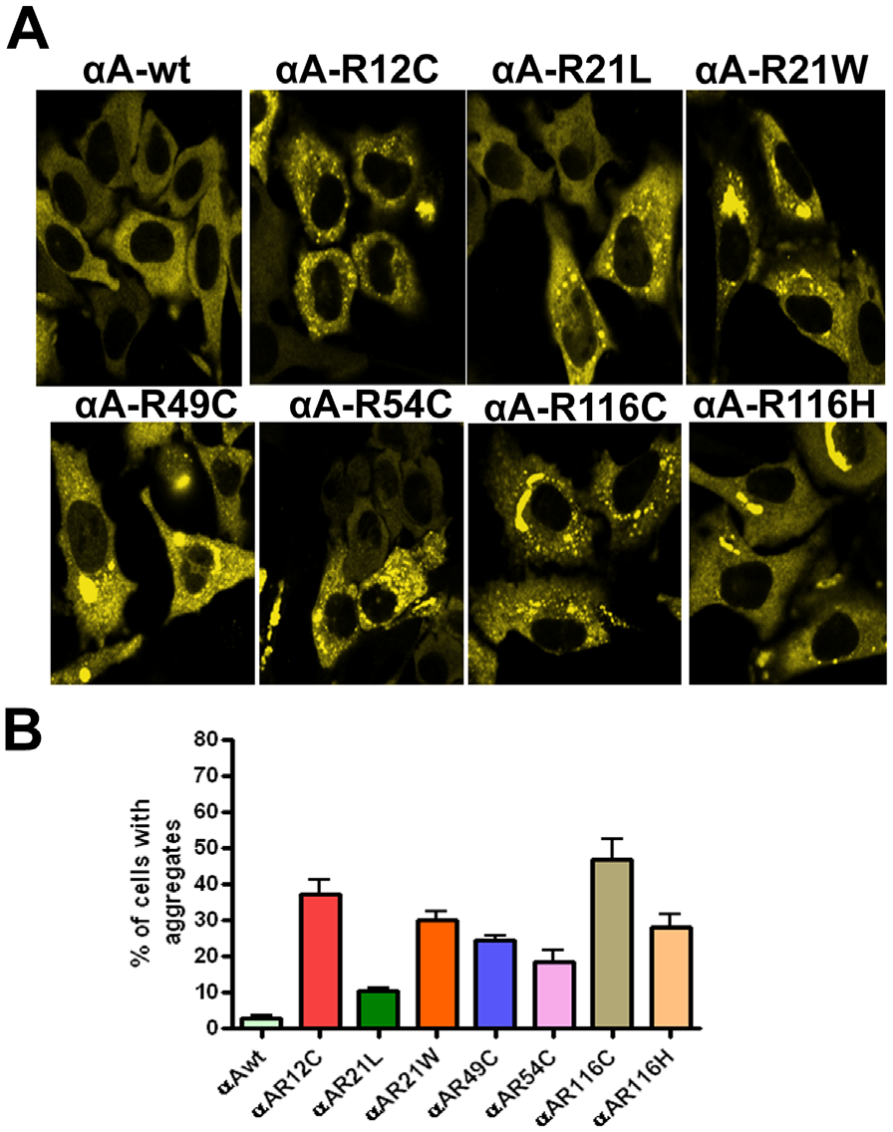
YFP-αA-wt and the mutant constructs, R12C, R21L, R21W, R49C, R54C, R116C and R116H were individually expressed in HeLa cells. *A: LSM Images were captured after 48 hours transfection.* HeLa cells were individually transfected with 2 µg of YFP-tagged αA-wt and mutants of αA-crystallin. A homogenous expression of αA-crystallin was evident in αA-wt transfected cells. Cytoplasmic aggregates were evident in αA- crystallin mutants transfected cells. The YFP signal was excited at 514 nm and the images were collected by BP 530-600 nm filter. The images represent one of the four similar images obtained in three independent experiments. *B: Graph represents per cent of cells with aggregates.* The results obtained after 48 hours transfection for the individually expressed αA-wt or its mutants in HeLa cells. Cells containing aggregates were counted in 10 random fields each field with 30 cells. The mutant, R116C showed a high per cent (∼47) of cells having aggregates and the mutant R21L showed least per cent (∼10) of cells containing aggregates. The results were presented as means ± SD obtained in three independent experiments. All the mutants were statistically significant, p < 0.01.

To investigate whether the mutants of αA-crystallin forms aggregates in cells, YFP-tagged wild-type and the mutants of αA-crystallins were transfected individually in HeLa cells. Cells transfected with CFP or YFP alone showed a homogenous expression of the fluorescent protein in both nucleus and cytoplasm (data not shown). Cells transfected with αA-wt showed a homogenous distribution of its expression in the cytoplasm alone and there was a little or no aggregation was observed in these cells (Fig. 1A). Cells transfected with the mutants, R12C, R21L, R21W, R49C, R54C, R116C, R116H showed significant number of cells having protein aggregates, 37.2±3.8, 10.5±2.6, 30.2±4.7, 24.5±1.1, 18.5±3.1, 47±5.8, 28.1±3.4 (Fig. 1B) respectively. Here, the intracellular aggregation is referred to as the clumped particles predominantly localized in the cytoplasm. Cells containing more than three such particles were considered as positive for cells having aggregates and scored in this assay. Moreover, the morphology of cells was altered in cells expressing R21W and R116C mutants (Fig. 1A).

### Co-expression of CFP-αA-wt and YFP-tagged mutants of αA-crystallins

**Figure 2:**
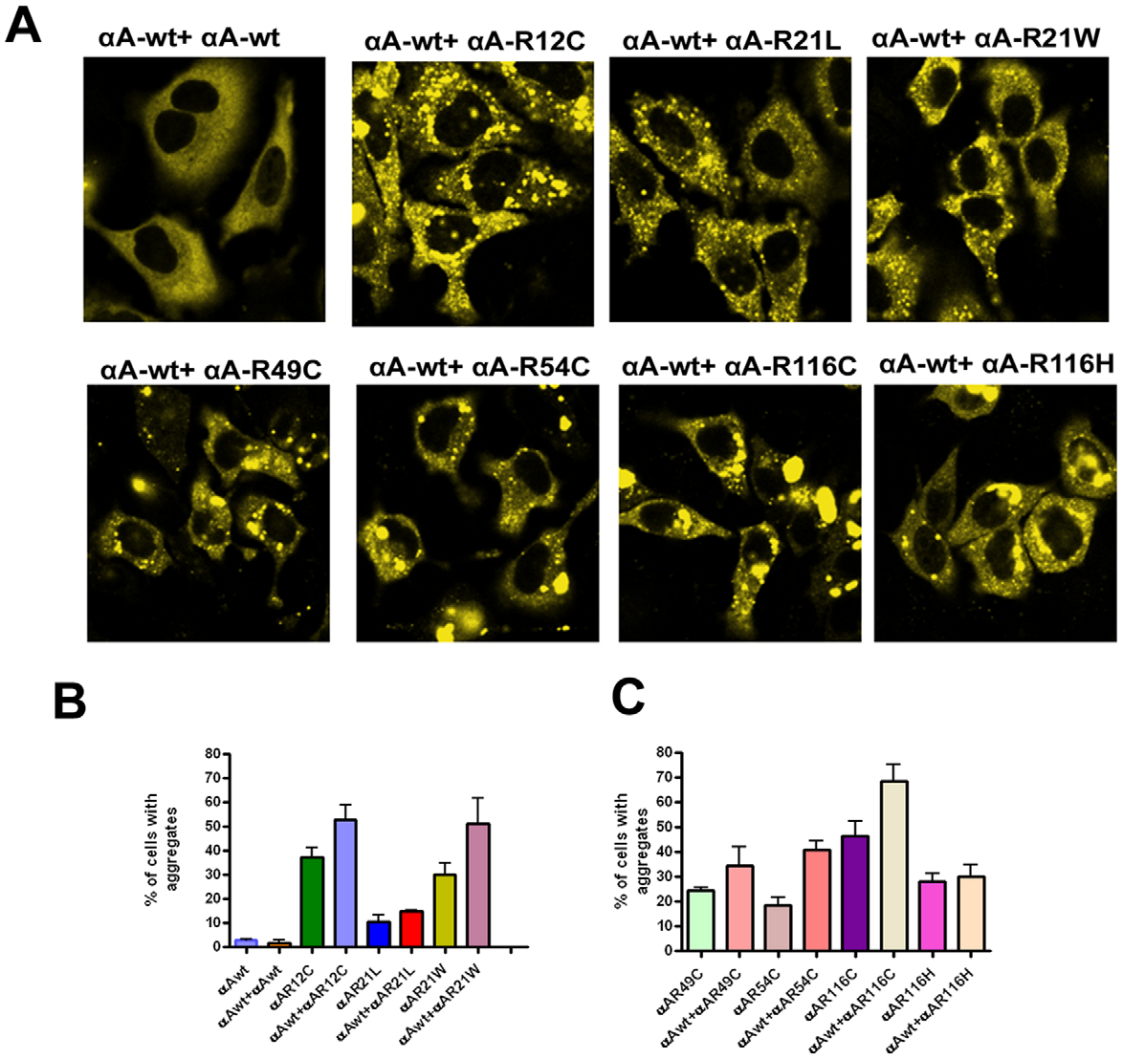
YFP-αA-wt and its mutants were co-expressed with CFP-tagged αA-wt. *A: Laser scanning confocal microscope images.* HeLa cells were transfected with 1 µg each of CFP-tagged αA-wt and YFP-tagged mutant constructs. After 48 h transfection, cells were analyzed with confocal microscope. Cells showed more aggregates when co-expressed with αA-wt. The CFP signal was excited at 458 nm and the images were collected by BP 475–525 nm filter, YFP was excited at 514 nm and the images were collected by BP 530–600 nm filter. The images represent one of the four similar images obtained in three independent experiments. *B&C: Graph represents per cent of cells with aggregates.* Cells were transfected with YFP-tagged αA-wt or mutants and CFP-tagged αA-wt. Cells having aggregates were counted in 10 random fields each field with 30 cells after 48 h transfection. The results were presented as means ± SD obtained in three independent experiments. For αA-wt + αA-R12C: p < 0.03; for αA-wt + αA-R21L : < 0.05; for αA-wt + αA-R21W : < 0.04, for αA-wt + αA-R49C, is not significant; for αA-wt + αA-R54C : < 0.0001; for αA-wt + αA-R116C : < 0.01 and for αA-wt + αA-R116H, is not significant.

To investigate whether co-expression of αA-wt can inhibit aggregates caused by mutants of αA-crystallin in cells, we transfected YFP-tagged αA-mutants with CFP-tagged αA-wt. Co-expression of CFP-αA-wt with YFP-αA-wt constructs in HeLa cells did not show any aggregates and a homogenous expression of protein was evident in the cytoplasm (Fig. 2A). However, in αA-wt co-expression with the mutants, R12C and R54C showed a few nuclear foci. Cells co-expressed with all the other mutants except R21L and R116H significantly increased the number of cells having aggregates. The proportion the cells with aggregates were 53, 15, 51, 35, 40, 68 and 30% in mutants, R12C, R21L, R21W, R49C, R54C, R116C and R116H respectively (Fig. 2B&C). Cells co-expressed with R12C, R49C and R116C distorted the cellular morphology (Fig. 2A). It is possible that the increase in the percent of cells having aggregates in the mutants expressing cells was due to co-aggregation with native αA-wt.

### Co-expression of CFP- tagged αB-wt and YFP-tagged mutants of αA-crystallin

**Figure 3:**
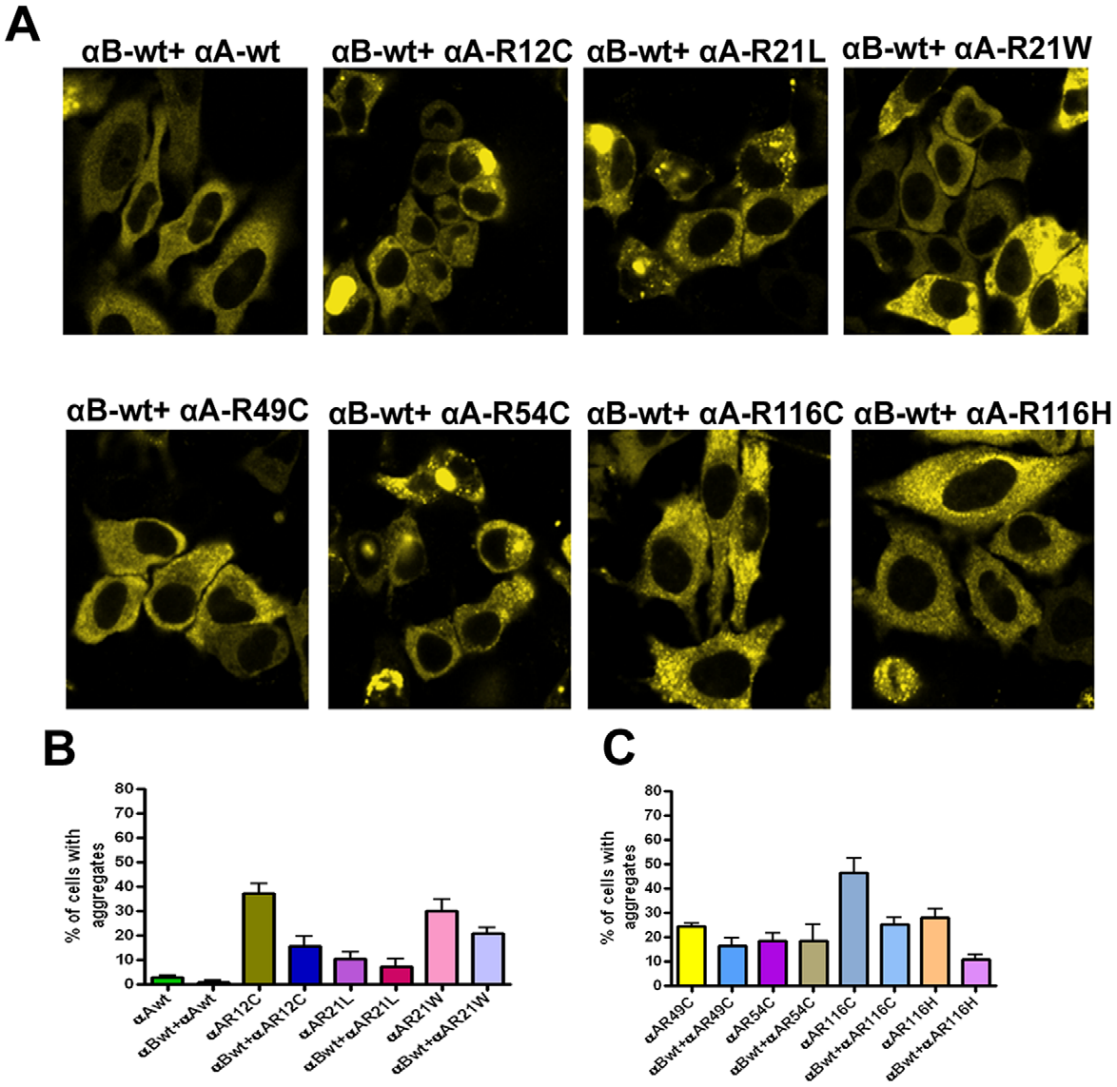
YFP-tagged αA-wt and each of the mutated αA-crystallin co-expressed with CFP-αB-wt. *A: LSM images of HeLa cells after 48 h transfection*. HeLa cells were transfected with 1 µg each of CFP-tagged αB-wt and YFP-tagged αA-wt and the mutants. After 48 h transfection, images were captured with an LSM confocal microscope. The CFP signal was excited at 458 nm and the images were collected by BP 475–525 nm filter, YFP was excited at 514 nm and the images were collected by BP 530–600 nm filter. Co-expression of αB-wt significantly inhibited aggregates in cells transfected with the αA- mutants. Images represent one of the four similar images obtained in three independent experiments. *B&C: Graph represents per cent of cells containing aggregates.* The results obtained after 48 h transfection. Cells containing aggregates were counted in 10 random fields each field with 30 cells. In all mutants, co-expression of αB-wt except with R21L and R54C significantly decreased the number of cells having aggregates. The results were presented as means ± SD obtained in three independent experiments. The p value for αB-wt + αA-R12C is < 0.02; for αB-wt + αA-R21L is not significant; for αB-wt + αA-R21W is < 0.05; for αB-wt + αA-R49C is < 0.02; for αB-wt + αA-R54C is not significant, for αB-wt + αA-R116C is < 0.01 and for αB-wt + αA-R116H is < 0.001.

To investigate, whether αB-wt co-expression can inhibit protein aggregates caused by mutants of αA-crystallin, cells were transfected with CFP-αB-wt and YFP-tagged αA-crystallin mutants.. The results indicate that a significant decrease (52–72%, as compared to the data in Fig. 2) of the number of cells having aggregates was observed in cells transfected with all the mutants but it is not statistically significant with the mutants, R21L and R54C. Specifically, the mutants, R12C, R21W, R116C and R116H showed the largest effect (61–72%) (Fig. 3A, B, C). Thus, co-expression of αB-wt with the mutants decreases protein aggregates. These results strongly suggest that αB-wt is a potential chaperone to protect the cells from aggregates caused by the αA-mutants.

### Validation of the expression of CFP or YFP-tagged αA-wt and/or αB-wt with mutants of αA-crystallin in HeLa cells

**Figure 4:**
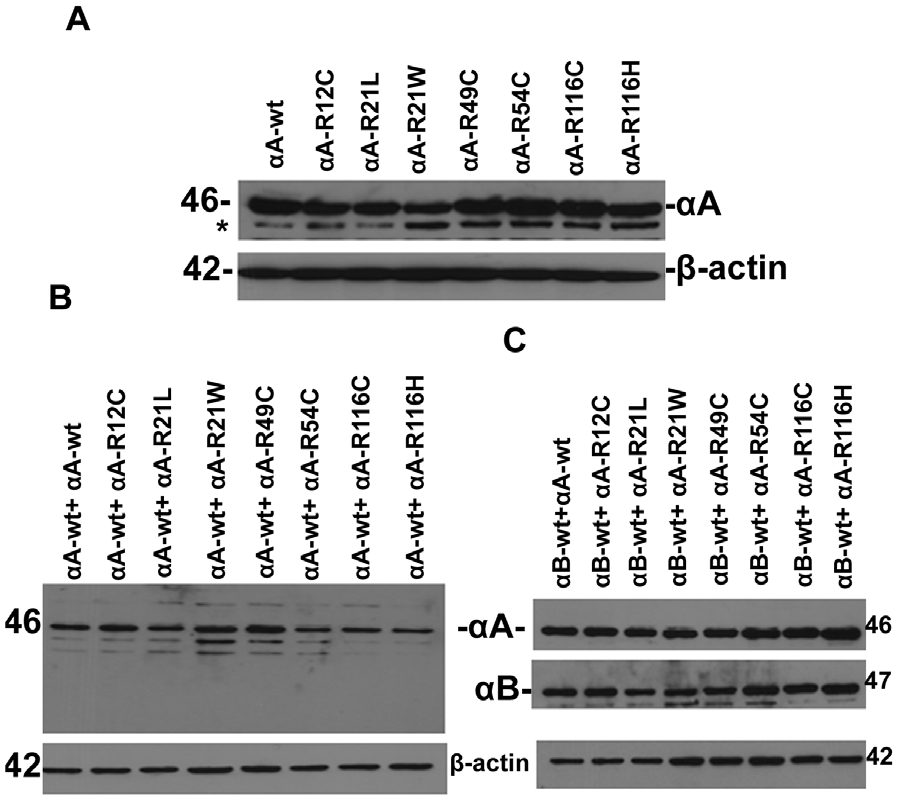
Western blot analysis of αA-crystallin-wt and its mutants, R12C, R21L, R21W, R49C, R54C, R116C and R116H expressed in HeLa cells. *A. Western blot analysis of HeLa cells individually expressed with αA-Crystallin:* Cells were individually transfected with YFP-tagged αA-wt and mutants. After 48 h transfection, cells were lysed and 5 µg of total protein was subjected to western blot. The blot was probed with human anti-αA antibody. The same blot was stripped and re-probed for β- actin to serves as a loading control. Nearly a similar level of expression of αA was evident in each of the transfected cells. The * indicates the non-speficific band. *B: Western blot analysis of HeLa cells co-expressed with CFP-tagged αA-wt and YFP-tagged αA-wt or its mutants*. Cells were co-transfected with YFP-tagged αA-wt and mutants with CFP-tagged αA-wt. After 48 h transfection, cells were lysed and 5 µg of total protein was subjected to western blot. The blot was probed with an antibody against human αA and the same blot was stripped and re-probed for actin. A similar level of expression of αA was detected in each group. *C: Western blot analysis of HeLa cells co-expressed with CFP-tagged αB-wt and YFP-tagged αA-wt or its mutants*. After 48 h transfection, 5 µg of total protein was subjected to western blot. The blot was probed with an antibody against human αA and the same blot was stripped and re-probed for anti-αB. The same blot was again stripped with anti-β-actin for loading control. The level of both αA and αB were nearly equal in each of the transfected cells.

To validate that there was no discrepancy in the transfection efficiency of the either individually transfected or co-transfected with αA-wt and or αB-wt, total cell lystate were subjected to immunoblot and probed with αA and or αB-crystallin antibodies. A similar level of expression was detected in individually expressed αA-wt or mutant constructs (Fig. 4A). The level of expression was nearly equal in mutants co-expressed with αA-wt (Fig. 4B) and αB-wt constructs (Fig. 4C).

### Mutant αA-crystallins in HeLa cells form aggresomes

**Figure 5:**
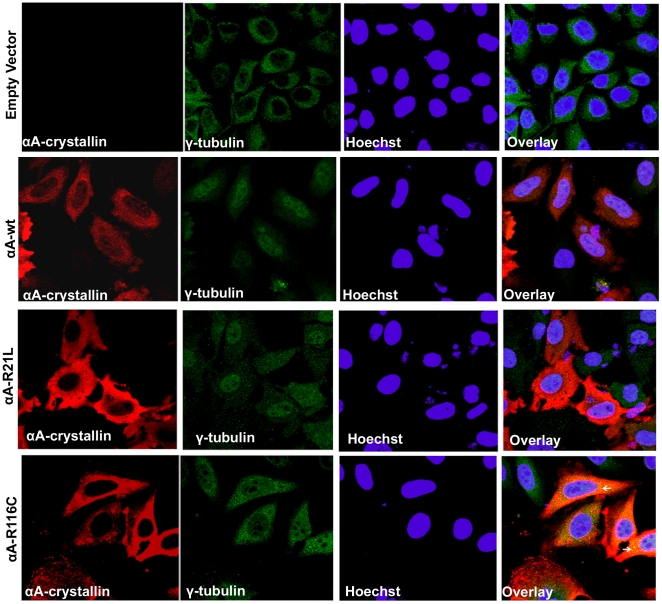
Detection of Aggresomes: Untagged pCDNA3.1 constructs of αA-wt and the mutants, R21L and R116C were used in this study and compared. After 48 h transfection, cells were fixed, permeabilized with 0.5% Triton X-100 and double immunostained with a mouse monoclonal αA-crystallin (red) antibody and a rabbit polyclonal antibody for γ-tubulin (green). A strong degree of overlapping signal (yellow) was evident only in R116C transfected cells (arrow). Co-localization of γ-tubulin, a centrosome maker protein with αA-crystallin in the perinuclear region validated these inclusions were aggresomes. There was no co-localization in cells transfected with either αA-wt or the mutant, R21L. Goat anti-mouse Alexa Fluor 594 (red) antibody was used to stain and visualize the localization of αA-wt and the mutants, R21L and R116C. A rabbit secondary antibody, Alexa Fluor 488 (green) was used to stain and visualize the γ-tubulin. The nuclear stain Hoechst was used to counter stain the nuclei. The images were representative of one of four such images obtained in three independent experiments.

To demonstrate whether the large perinuclear structures are aggresomes as seen in the cells transfected with the mutants, R21W, R116C and R116H (three major aggregate forming mutants) and to validate these inclusions as aggresomes, transfected cells were subjected to double immunostaining with a mouse monoclonal αA-crystallin (red) and rabbit polyclonal γ-tubulin, (a centrosome marker protein) (green) antibodies. For this study, untagged pCDNA3.1 constructs of both wild-type and the mutants R116C (severely affected cells) and R21L (mildly affected cells) were used in order to eliminate the false positive signal by overlapping of YFP signal with the Alexa Fluor 488, both of these signals being acquired at argon-ion laser line in confocal microscopy. The results showed that only in R116C, the co-localization of αA-crystallin with γ-tubulin occurred as yellow punctate signals in the perinuclear region validated these structures are aggresomes (Fig. 5) whereas, there were no aggresomes in R21L expressing cells (Fig. 5).

### Degradation of aggregate-prone αA-crystallin mutants

**Figure 6:**
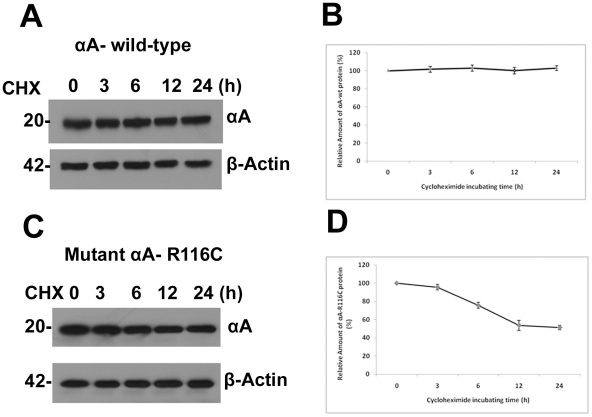
Degradation of aggregate-prone αA-crystallin mutant, R116C. *A and C: Western blot analysis of cycloheximide treated cells:* HeLa cells were transfected with 2 µg of untagged pCDNA3.1 constructs of αA-wt and the mutant, R116C. After 24 h transfection, cells were treated with 20 µg/ml of cycloheximide and lysed with lysis buffer at indicated time points. For each of the sample, 5 µg of total protein was loaded and western blot probed with an anti-αA-crystallin (rabbit polyclonal, Enzo Life Sciences Inc., Catalog # SPA-221). The mutant R116C protein level has decreased after 6 hours treatment with cycloheximide compared to wild-type protein which demonstrated R116C protein instability. The β-Actin blot serves as a loading control. *B and D: Quantification of wild-type and the mutant, R116C protein at different time points.* The density of the band was quantified using NIH Image J software and plotted. Values represent as means ± SD as obtained in three independent experiments. The mutant, R116C Vs wild-type control is significant at p < 0.001.

Since our finding that mutants of αA-crystallin form intracellular aggregates in cells and this aggregate formation may affect protein turn over which may contribute to the pathogenesis of the cataract, we next asked whether mutation in αA-crystallin may affect protein's turn over by faster degradation, cells were treated with cycloheximide (for inhibition of protein synthesis) and at different time points. After 24 h transfection, the cells lysed and a total protein of 5 µg from each of the sample was subjected to immunoblot probed with αA-crystallin antibody (rabbit polyclonal, Enzo Life Sci, SPA-221). For this study, we used untagged constructs of αA, i.e. pCDNA3.1/αA-wt and pCDNA3.1/αA-R116C. As shown in Fig. 6A and Fig. 6B, pulse chase experiments followed by cycloheximide treatment showed that wild-type protein is stable at least for 24 h. But the level of the mutant protein R116C decreased to 75.6±3.2%, 53±5.5%, and 51.3±2.5% compared to control after 6, 12 and 24 h of treatment respectively (Fig. 6C and 6D) As expected the mutant protein has lower half-life and degraded faster than the wild-type protein.

### Accumulation of polyubiquitinated conjugates in αA-crystallin mutants

**Figure 7:**
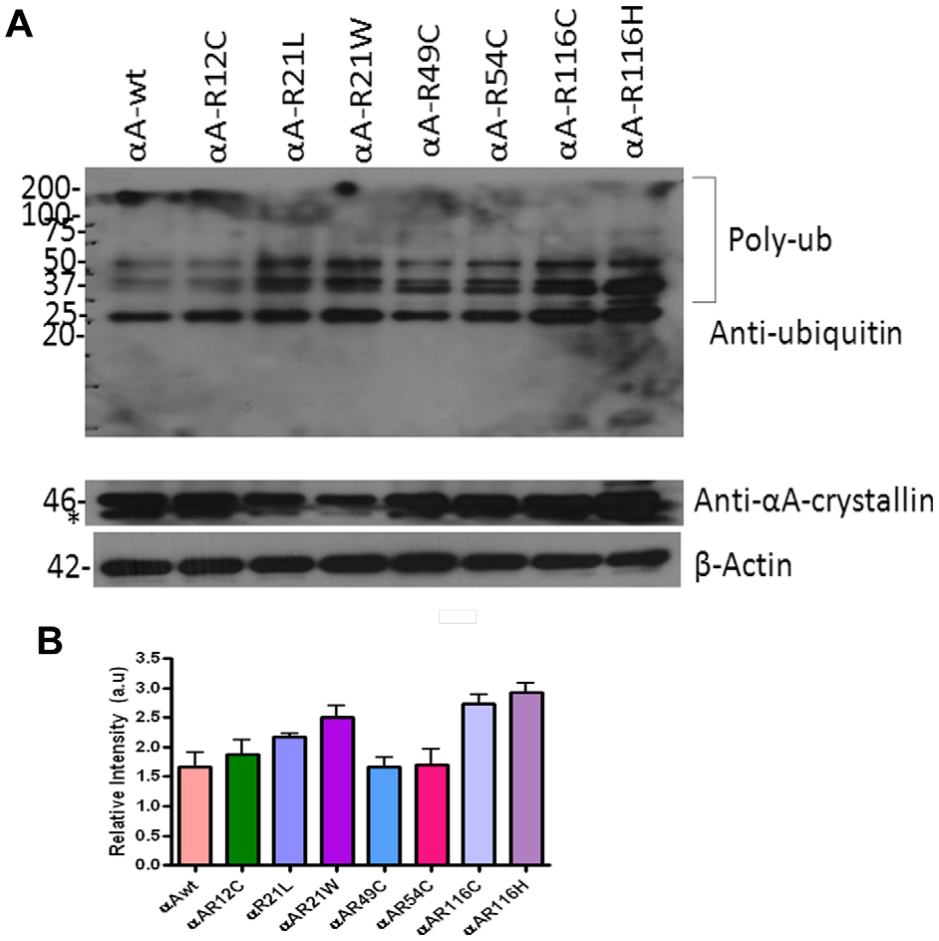
Overexpression of αA-Crystallin mutants showed increased ubiquitination. *A: Western blot analysis:* After 48 h transfection, cells were lysed and 5 µg of total protein was subjected to immunoblot probed with an anti-ubiquitin (FK2) monoclonal antibody. The pattern of polyubiquitinated species were dramatically increased in the mutants, R21W, R116C and R116H compared to other mutants and wild-type. * indicates a non-specific band. The same blot was stripped and re-probed for αA-crystallin and β-actin for loading controls. The blot shown here is a representative blot of three independent experiments. *B: Quantitative data for the western blot:* Using NIH Image J software, densitometric measurements were normalized against β-actin. The mutants, R21L, R21W, R116C and R116H showed increased ubiquitination as obtained in three independent experiments and plotted. The results were expressed as mean ± SD. The p value for αA-wt Vs αA-R12C is not significant, for αA-wt Vs αA-R21L is < 0.05; for αA-wt Vs αA-R21W is < 0.01; for αA-wt Vs αA-R49C is not significant; for αA-wt Vs αA-R54C is not significant; for αA-wt Vs αA-R116C is < 0.003 and for αA-wt Vs αA-R116H is < 0.001.

**Figure 8:**
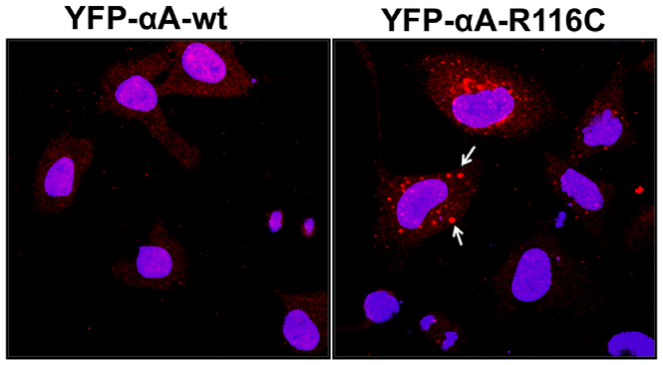
Overexpression of αA-crystallin mutants shows accumulation of ubiquitin inclusions in HeLa cells. YFP-tagged αA-wt and mutant, R116C were transfected in HeLa cells. After 48 h transfection, cells were fixed and immunostained with a mouse monoclonal ubiquitin (FK2) antibody and further stained with a fluorescent conjugated secondary antibody, Alexa Flour 594 (red). The arrows indicate ubiquitinated cytoplasmic inclusions only in the mutant, R116C expressing cells. Hoechst 33342 was used to counter stain the nuclei. The images were representative of three similar images obtained in three independent experiments.

A number of reports implicated UPS dysfunction in a range of aggregation prone mutant proteins in neurodegenerative diseases. To explore any effects of aggregation-prone αA-crystallin mutants might have on UPS proteolytic function, we measured the level of polyubiquitinated proteins in whole cell lysate subjected to western blot probed with anti-ubiquitin antibody (FK2, Mouse Monoclonal, Enzo Life Sciences, PW8810). Distinct polyubiquitin conjugated proteins accumulated in cells transfected with mutants, R21L, R21W, R116C and R116H (Fig. 7, lanes 3, 4, 7 and 8). In a separate experiment, to further show ubiquitin inclusions in transfected cells, cells were subjected to immunostaining with a mouse monoclonal ubiquitin antibody (FK2; Enzo Life Sci.Inc., PW 8810). The ubiquitin inclusions in the perinuclear regions were evident only in the mutant R116C transfected cells (Fig. 8). Altogether, these results suggest that mutants of αA-crystallin proteins were conjugated with ubiqutin for degradation.

## Discussion

In the present study, we have demonstrated that overexpression of the cataract causing mutants of αA-crystallin in HeLa cells led to the formation of multiple intracellular protein aggregates. There was no evidence for the endogenous expression of both αA- and αB-crystallins in these cells as shown in our earlier study [19] and thus serves as a perfect model to study the role of cataract causing mutants of αA-crystallin in mammalian cells including the eye lens cells. Compared to αA-wt, expression of all the mutants in these cells showed significant increase in protein aggregation after 48 hours of transfection, R21L showing the least increase although higher than the control αA-wt. It is likely that the protein aggregation in the cytoplasm was due to protein conformational changes the mutants undergo [11]. When these mutants were expressed individually, they formed aggregates probably due to stress in the absence of any protective mechanism such as the presence of αB-crystallin, a powerful sHsp. sHsps, in general, can inhibit protein aggregation and can reverse or refold aggregated proteins in conjunction with Hsp 70, a major molecular chaperone. As shown in an earlier study of the C-terminal truncated αA-crystallins [19], association with native αA-crystallin significantly increased the number of cells containing aggregates in all mutants. These results suggest that αA-crystallin is not a potential chaperone to protect the cells from protein aggregation. It is interesting to note that most of the mutants of αA-crystallin involve arginine residues and lead in a dominant fashion. Co-expression with αB-crystallin, on the other hand, significantly diminished the aggregation, R12C, R21L_,_ R116C and R116H showing the most effect and R49C and R54C showing the least effect. αB-crystallin is known to be a better molecular chaperone than αA-crystallin and as it has been shown earlier with C-terminal truncated αA-crystallins [20], it readily recognizes partially unfolded structures and prevent them from aggregation.

Also, this study provides the first evidence for cataract causing mutants of αA-crystallin forming aggresomes in cells. Accumulation of misfolded proteins results from saturation of protein degradation system observed in conformational diseases like Huntington disease [21] and cystic fibrosis [22] leading to the formation of inclusion bodies also known as aggresomes. The inclusion bodies concentrated in the perinuclear region of αA-crystallin mutants expressed in HeLa cells suggests that they have the characteristic features of aggresomes. They are cytoplasmic globular structures formed due to protein misfolding in the cytosol and these structures are delivered to the microtubule organizing center (MTOC) by retrograde transport along microtubules [12]. These aggresomes are not merely a storage site for misfolded proteins; they can facilitate the degradation of protein aggregates [12] and are the pathological hallmark of conformational diseases that results from protein misfolding. In the present study, double immunofluorescence results (Fig. 5) validate the co-localization of αA-crystallin with a centrosome marker protein, γ-tubulin, in aggresomes. The γ-tubulin has been previously shown to co-localize with aggresomes in MTOC [23]–[25]. They are normally formed when ubiquitin-proteasome degradation pathway is impaired and disease-associated proteins inefficiently fold [26], [27]. Our findings on aggresomes detection in cells transfected with αA-crystallin mutants are very similar to the previous studies on a myopathy causing mutant of human αB-crystallin, R120G, which forms aggresomes in CCL39 cells [28]. Aggresomes are special protective structures that fundamentally differ from other multiple aggregates, that some of which cause cellular toxicity. They are formed around MTOC, a sub-cellular region which is robustly enriched with chaperones and components of UPS [12]. It has been reported that multiple aggregates or pre-aggresome particles may be an intermediate step in aggresome formation which can proceed further upon proteasome inhibition [15]. The present study documented both multiple aggregates and typical perinuclear localized aggresomes. Typical aggresomes were detected only in R116C mutant but not in R21L, which suggest that the multiple aggregates in cells expressing this mutant did not develop as aggresomes.

It has been suggested that unfolded proteins develop into insoluble form that cannot be degraded, their sequestration in one large mass may facilitate their removal by autophagy [23]. Degradation of C-terminal truncated αA-162 through ubiquitin-proteasome pathway has been reported previously using recombinant proteins [29]. Proteins with short half-lives are mostly degraded by the proteasome [30]. Our results on the mutant-R116C protein degradation upon cycloheximide treatment are consistent with a previous report [31] on a truncated αB-crystallin, 450delA protein which has lower half-life [31]. The Ubiquitin- Proteasome System (UPS) degrades short-lived nuclear, misfolded cytosolic proteins extruded from the endoplasmic reticulum [16]. Substrates of UPS need to unfold to pass through the narrow barrel-shaped proteasome which makes aggregate-prone proteins poor substrates for UPS [32], [33]. The pattern of ubiquitination as we documented in overexpression of three mutants of αA-crystallin, R21W, R116C and R116H suggest involvement of Ubiquitin Proteasome Pathway (UPP) in the turnover of ubiquitinated adducts even though other cellular machineries could also be engaged in this event. Although there was a basic degradation pattern evident in αA-wt but the intensity of the degraded bands at 25 and 37 kDa was stronger in the mutants compared to αA-wt. From this perspective, involvement of αA-crystallin in ubiquitination pathway may provide new insights into the etiology of congenital cataracts.

In summary, we have demonstrated overexpression of αA-crystallin mutants forming small dispersed cytoplasmic aggregates and aggresomes in HeLa cells. Co-expression of αB-wt significantly inhibits aggregates caused by the αA mutants. The mutant R116C has short half-life and degraded through ubiquitin-proteasome pathway that may contribute to the development of congenital cataract in human beings.
